# Carbohydrate counting knowledge and its association with diabetes distress in patients with type 1 diabetes: a cross-sectional study from the United Arab Emirates

**DOI:** 10.3389/fendo.2026.1922652

**Published:** 2026-08-26

**Authors:** Adnan Agha, Sozan Yasser Rakha, Bachar Afandi, Nishan Sudheera Kalupahana, Juma M. Alkaabi

**Affiliations:** 1 Department of Internal Medicine, College of Medicine and Health Sciences, United Arab Emirates University, Al Ain, United Arab Emirates; 2 Department of Nutrition and Health, College of Medicine and Health Sciences, United Arab Emirates University, Al Ain, United Arab Emirates; 3 Department of Endocrinology, Tawam Hospital, Al Ain, United Arab Emirates

**Keywords:** AdultCarbQuiz, carbohydrate counting, diabetes distress, hypoglycaemia awareness, type 1 diabetes mellitus, United Arab Emirates

## Abstract

**Introduction:**

Carbohydrate counting is central to dietary self-management in patients with type 1 diabetes mellitus (T1DM). However, objective assessments of carbohydrate counting knowledge and its relationship with psychosocial outcomes are scarce in Gulf populations. This study assessed carbohydrate counting knowledge, diabetes distress, and hypoglycaemia awareness in patients with T1DM attending a tertiary diabetes centre in the United Arab Emirates.

**Methods:**

This was a cross-sectional study of 40 patients with T1DM (age ≥ 14 years, diabetes duration ≥ 12 months) attending the Tawam Hospital Diabetes Clinic, Al Ain, UAE. Carbohydrate counting knowledge was assessed using the Arabic version of the 43-item AdultCarbQuiz questionnaire. Diabetes distress was measured using the two-item Diabetes Distress Scale (DDS-2), and hypoglycaemia awareness was measured using the Gold score. Pearson’s correlations, one-way analysis of variance (ANOVA) across knowledge quartiles, and partial correlations adjusted for HbA1c and Gold score were performed.

**Results:**

The mean AdultCarbQuiz score was 26.2 ± 4.0 out of 43 (60.9%). Domain analysis revealed strong carbohydrate food recognition (domain mean 80.1% correct) but poor quantification skills (20-27.5% correct for portion estimation) and meal-level counting (2.5-15% correct). Diabetes distress was present in 37.5% of the participants, and impaired hypoglycaemia awareness was present in 20.0% of the participants. Knowledge of carbohydrate counting showed a moderate inverse correlation with diabetes distress (r = -0.468, 95% CI -0.68 to -0.18, p = 0.002), which strengthened further after adjusting for HbA1c and Gold score (partial r = -0.579, 95% CI -0.77 to -0.30, p < 0.001). In exploratory analyses, younger age (r = -0.404, p = 0.010) and shorter diabetes duration (r = -0.344, p = 0.030) were associated with higher knowledge scores. No association was observed between the knowledge scores and HbA1c levels (r = 0.179, p = 0.295).

**Discussion:**

In this cross-sectional sample, carbohydrate counting knowledge was independently and inversely associated with diabetes distress, regardless of glycaemic control. Specific knowledge gaps in carbohydrate quantification and meal-level counting are actionable targets for educational intervention in this population. These findings highlight the value of addressing both nutritional knowledge and emotional well-being in T1DM management; whether improving carbohydrate-counting knowledge reduces distress requires longitudinal and interventional study.

## 1 Introduction

Carbohydrate counting is an established meal-planning strategy that enables patients with type 1 diabetes mellitus (T1DM) to match their prandial insulin doses to their dietary carbohydrate intake (1, 2). The Diabetes Control and Complications Trial demonstrated that intensive insulin therapy, incorporating flexible dietary management, including carbohydrate counting, reduced the risk of microvascular complications by 34-76% (3). Meta-analyses of randomised controlled trials have also confirmed that structured carbohydrate counting education reduces HbA1c by 0.5-0.7% compared with standard dietary advice, without increasing hypoglycaemia or body weight in patients with T1DM (4, 5). Therefore, current international guidelines endorse carbohydrate counting as the recommended nutritional approach for patients with T1DM undergoing intensive insulin therapy (6).

Despite this evidence base, the practical implementation of carbohydrate counting in clinical settings depends on patients possessing adequate knowledge to identify carbohydrate-containing foods, estimate portion sizes, quantify the carbohydrate content in individual foods, interpret nutrition labels, and to be able to apply this information to a variety of meals (7). Published data from cross-sectional studies indicate that carbohydrate counting knowledge is often suboptimal, with meal-level carbohydrate estimation errors averaging 15-28% (8). These errors predict greater glycaemic variability and may undermine the benefits of flexible insulin dosing in patients with T1DM (8, 9). Therefore, assessing carbohydrate counting knowledge using validated instruments is critical for identifying educational gaps and tailoring diabetes self-management education programs.

The AdultCarbQuiz is a 43-item self-administered questionnaire developed by Watts et al. (7) to assess carbohydrate counting knowledge across six domains: carbohydrate food recognition (19 items), quantification of carbohydrates in single foods (seven items), nutrition label interpretation (four items), glycaemic targets (four items), hypoglycaemia prevention and treatment (five items), and meal-level carbohydrate counting (four items). The instrument demonstrated good reliability (Kuder-Richardson 20 coefficient = 0.90) and clinical validity in the original American cohort (7). Cross-cultural adaptations have been completed in Arabic for the Saudi population (10) and in Portuguese (11), with acceptable psychometric properties and demonstrated associations between quiz scores and glycaemic control.

In the Middle East and North Africa (MENA) region, an estimated 55 million adults live with diabetes, with approximately 150,000 children and adolescents affected by T1DM (12, 13). Bawazeer et al. reported a mean AdultCarbQuiz score of 23.0 ± 7.3 out of 43 (53.5%) amongst 224 Saudi adults with T1DM, with carbohydrate counting usage, frequency of education, insulin pump therapy, and lower HbA1c identified as predictors of higher scores (10). An earlier pilot study of 94 Emirati and Omani adults with diabetes reported low knowledge of carbohydrate foods but did not employ a validated assessment instrument (14). To our knowledge, no prior study has assessed carbohydrate counting knowledge using a validated tool in a UAE population with T1DM.

An emerging body of evidence links diabetes-related distress to poor self-management behaviours and reduced perceived competence in T1DM (15, 16). Distress, distinct from clinical depression, reflects the emotional burden of living with a demanding chronic condition and affects 20-40% of adult patients with T1DM (17). Cross-sectional studies have demonstrated inverse associations between diabetes distress and self-management competence (15), and longitudinal data from the T1-REDEEM trial showed that reductions in diabetes management distress were associated with improvements in self-care skills, including the perceived ability to adjust insulin to dietary intake (16). However, whether a specific, measurable association exists between carbohydrate counting knowledge and diabetes distress, independent of glycaemic control, has not yet been examined.

This study assessed carbohydrate counting knowledge using the Arabic AdultCarbQuiz in patients with T1DM who attended a tertiary diabetes centre in the UAE. The primary objective was to determine the association between carbohydrate counting knowledge and diabetes distress, independent of glycaemic control and hypoglycaemia awareness in these patients with T1DM. The secondary objectives of our study included identifying domain-specific knowledge gaps and evaluating the demographic and clinical predictors of carbohydrate counting performance.

## 2 Methods

### 2.1 Study design and setting

This single-centre, cross-sectional study was conducted at the Diabetes Clinic of Tawam Hospital, a tertiary care facility in Al Ain, Abu Dhabi Emirate, UAE, with participant enrolment over a 16-month period from 1 November 2023 to 1 March 2025. The study was approved by the Tawam Hospital Research and Ethics Committee (approval number 955-THREC, dated 04/08/2023). Written informed consent was obtained from all participants; for those aged 14–17 years, parental or guardian consent was also obtained.

### 2.2 Participants

Consecutive patients aged ≥ 14 years with a confirmed diagnosis of T1DM for at least 12 months were invited to participate in this study during their routine clinic visits. All participants were on a basal-bolus insulin regimen or insulin-pump therapy. Patients who could not read or understand Arabic and those with cognitive impairments were excluded. Of approximately 200 patients with T1DM eligible and in active follow-up, 136 were approached during routine clinic visits over the study period, and 64 could not be approached within the recruitment window. Of the 136 approached, 96 declined and 40 consented and were enrolled, giving a response rate of 29.4% amongst those approached and representing 20% of the eligible source population. No approached patient was excluded on eligibility grounds.

### 2.3 Sample size considerations

This was an exploratory study. Formal *a priori* sample size calculation was constrained by the small source population of approximately 200 patients with T1DM followed at the clinic, and a pragmatic approach was adopted, enrolling 40 participants (approximately 20% of the eligible population). With this sample size, a two-sided alpha of 0.05, and using the Fisher z-transformation, the study had 80% power to detect a correlation of r ≥ 0.43 (minimum detectable r = 0.43). The study was therefore powered for the primary association only; secondary associations with smaller effect sizes were underpowered and are reported as exploratory and hypothesis-generating.

### 2.4 Data collection and instruments

Demographic and clinical data were recorded on a standardised proforma, including age, sex, nationality, diabetes duration, insulin regimen details, body mass index (BMI), HbA1c (most recent value within three months), lipid profile, renal function, and comorbidities. HbA1c was measured in the Tawam Hospital medical laboratory using a Roche Integra 400 Plus automated analyser (Roche Diagnostics), an NGSP-certified and DCCT-aligned method, and reported as a percentage. The following three validated instruments were administered.

**AdultCarbQuiz (Arabic version):** The 43-item self-administered questionnaire assesses carbohydrate counting knowledge across six domains (7, 10). Each item was scored as correct (1) or incorrect (0); ‘do not know’ responses were scored as incorrect. The maximum score was 43, with higher scores indicating greater knowledge. The Arabic version was translated and validated by Bawazeer et al., with acceptable reliability (Cronbach’s alpha 0.87); as this version uses Modern Standard Arabic, shared with the UAE, no separate local re-validation or *de-novo* pilot was undertaken before administration in this cohort (10).

**Diabetes Distress Scale-2 (DDS-2):** A two-item screening measure assessing feelings of being overwhelmed by diabetes (item 1) and perceived failure in diabetes management (item 2), each scored on a six-point Likert scale (1-6). An average score of ≥ 3 indicates the presence of diabetes distress (18, 19).

**Gold score:** A single-item seven-point scale assessing awareness of hypoglycaemia, where a score of 1 indicates full awareness and ≥4 indicates impaired awareness (20).

### 2.5 Statistical analysis

The distribution of the primary outcome (total correct score on the AdultCarbQuiz) was assessed using histograms, Q-Q plots, and the Shapiro-Wilk test; every continuous variable was likewise examined for normality. Continuous variables are summarised as mean ± SD, or as median [IQR] where skewed, and categorical variables as frequency (percentage). Pearson’s correlation coefficients, with 95% confidence intervals, examined linear associations between the carbohydrate counting score and continuous demographic, clinical, and psychosocial variables, and Spearman’s rank correlations were computed as a non-parametric sensitivity analysis. For descriptive comparison, participants were additionally stratified into three groups by total-correct-score quartile — lowest (score ≤24, n = 10), middle two quartiles combined (score 25-28, n = 17), and highest (score ≥29, n = 13; boundaries Q25 = 24.75, Q75 = 29.0); the continuous score remained the primary variable for all correlation analyses, so this categorisation did not affect the primary result. Between-group comparisons used one-way ANOVA, with Levene’s test for homogeneity of variance and Tukey HSD *post-hoc* tests following a significant result, and Welch ANOVA and Kruskal-Wallis tests where variances were unequal; categorical comparisons used Fisher’s exact test where expected cell counts were below five. Partial correlations assessed the independent association between carbohydrate counting knowledge and diabetes distress, adjusting *a priori* for HbA1c and Gold score as the principal clinical confounders (glycaemic control and hypoglycaemia awareness), with a sensitivity model additionally adjusting for age and diabetes duration. “Days since last carbohydrate-counting education” was recorded with “daily” coded as 0 days. Analyses were complete-case with no imputation; denominators are reported per analysis, and partial correlations involving HbA1c were computed on the 36 participants with an available value. Statistical significance was set at p < 0.05. All analyses were performed using Python (SciPy version 1.11) with cross-validation against manual calculations and SPSS version 28 (IBM Corporation, Armonk, NY, USA).

## 3 Results

### 3.1 Participant characteristics

**Figure 1:**
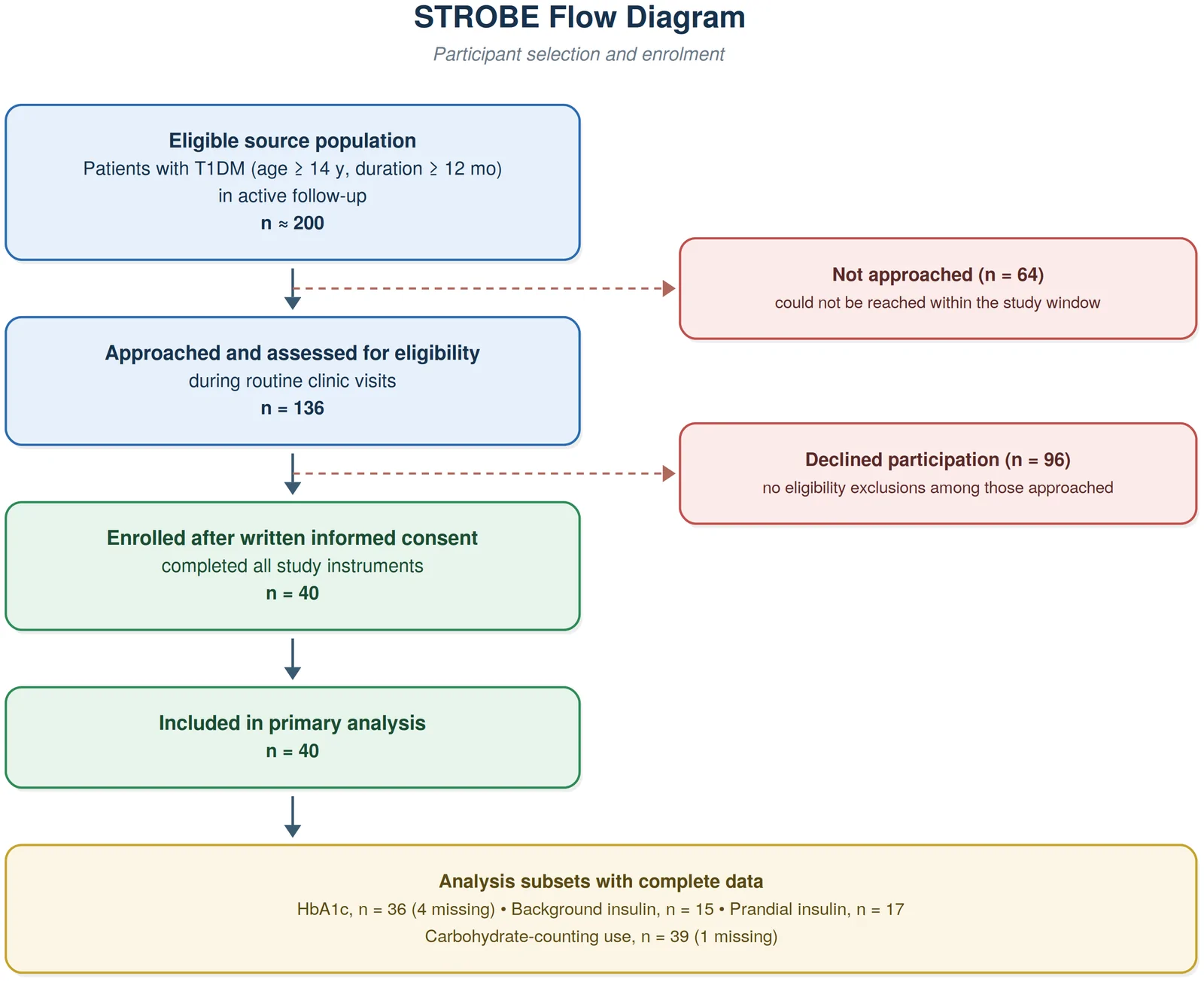
STROBE flow diagram of participant selection and enrolment. Of approximately 200 eligible patients with T1DM at Tawam Hospital Diabetes Clinic, 136 were approached (64 could not be approached within the recruitment window); of these, 96 declined and 40 were enrolled and included in the primary analysis. No approached patient was excluded.

A total of 40 patients with T1DM were included in the study. The participant flow is shown in **Figure 1**. The mean age was 24.0 ± 11.4 years, 25 (62.5%) were female, and 35 (87.5%) were Emirati nationals. The mean diabetes duration was 11.0 ± 9.0 years. The mean HbA1c was 8.1 ± 1.6% (n = 36), indicating suboptimal glycaemic control. The mean BMI was 26.0 ± 5.7 kg/m^2^. Most participants (87.5%) self-administered insulin. Of those with available data, 35 of 39 (89.7%) reported using carbohydrate counting for insulin dose adjustment. Details of sociodemographic and clinical characteristics of the patients are shown in **Table 1**.

**Table 1:** Sociodemographic and clinical characteristics of the study participants (n = 40).

Variable	n	Mean ± SD or n (%)	Median [IQR]
Demographic characteristics			
Age (years)		23.98 ± 11.42	20.00 [15.00–32.00]
Sex — female		25 (62.5%)	—
Sex — male		15 (37.5%)	—
Nationality — Emirati		35 (87.5%)	—
Nationality — non-Emirati		5 (12.5%)	—
Alcohol use/smoking — yes		2 (5.0%)	—
Alcohol use/smoking — no		38 (95.0%)	—
Clinical characteristics			
Diabetes duration (years)		11.05 ± 9.00	7.50 [4.75–17.25]
Body mass index (kg/m²)		26.00 ± 5.72	—
HbA1c (%)	36	8.06 ± 1.55	7.90 [7.15–8.62]
Total cholesterol (mmol/L)		4.60 ± 1.19	4.37 [3.84–4.91]
Triglycerides (mmol/L)		0.95 ± 1.22	0.71 [0.50–0.89]
HDL cholesterol (mmol/L)		1.60 ± 0.36	—
LDL cholesterol (mmol/L)	39	2.56 ± 1.05	2.61 [1.88–2.96]
Treatment-related characteristics			
Total daily insulin dose (U/day)		78.47 ± 40.51	70.00 [60.00–90.00]
Insulin administered by patient		35 (87.5%)	—
Insulin administered by parent/caregiver		5 (12.5%)	—
Psychosocial and knowledge measures			
DDS-2 average score (1–6)		2.35 ± 0.90	2.00 [2.00–3.00]
Diabetes distress (DDS-2 ≥ 3) — present		15 (37.5%)	—
Diabetes distress; absent		25 (62.5%)	—
Gold score (1–7)		2.38 ± 1.51	2.00 [1.00–3.00]
Impaired hypoglycaemia awareness; (Gold ≥ 4)		8 (20.0%)	—
Normal hypoglycaemia awareness (Gold < 4)		32 (80.0%)	—
AdultCarbQuiz total correct score (0–43)		26.20 ± 4.03	—

Data are mean ± SD for continuous variables and n (%) for categorical variables. Median [IQR] is additionally reported for variables with a skewed distribution (Shapiro–Wilk p < 0.05); a dash indicates that the variable was approximately normally distributed or categorical. The n column is completed only where data were missing; all other variables were complete for the full cohort (n = 40). BMI, body mass index; DDS-2, two-item Diabetes Distress Scale; HbA1c, glycated haemoglobin; HDL, high-density lipoprotein; LDL, low-density lipoprotein.

### 3.2 Carbohydrate counting knowledge

The mean total correct score on the AdultCarbQuiz was 26.2 ± 4.0 out of 43 (60.9%), with scores ranging from 16 to 33. The distribution was approximately normal (Shapiro-Wilk p = 0.053, skewness = -0.63, and kurtosis = 0.14), supporting the use of parametric methods.

**Figure 2:**
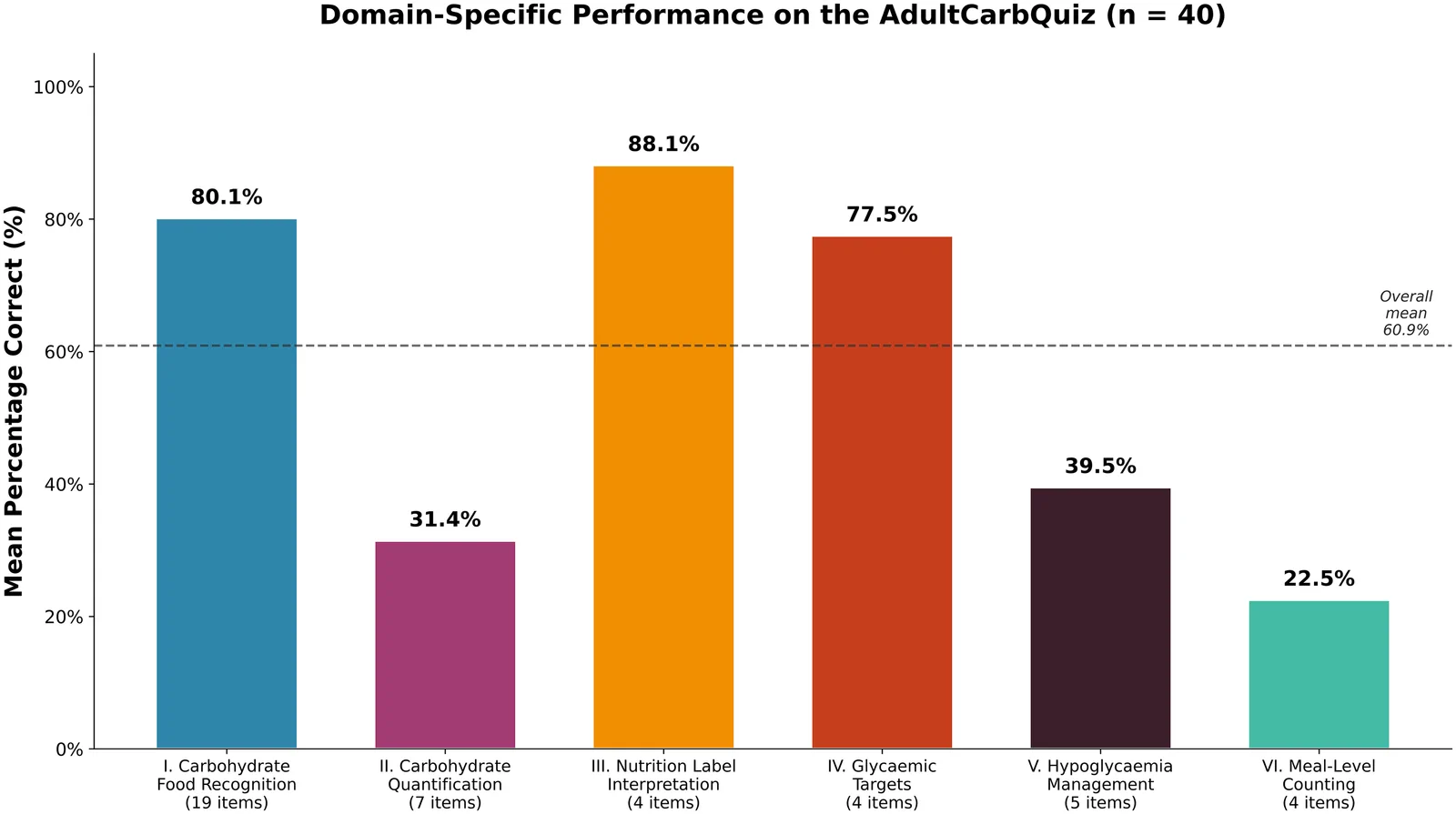
Domain-specific performance on the AdultCarbQuiz (n = 40). Bars represent mean percentage correct within each of the six domains. Dashed line indicates overall mean (60.9%).

Domain-specific analysis revealed a clear dissociation between carbohydrate recognition and quantification (**Figure 2**). In Domain I (carbohydrate food recognition), participants correctly identified common carbohydrate sources, such as bread (97.5%), apple juice (100%), and cooked rice (100%). The performance was weaker for foods with ambiguous carbohydrate status: butter was correctly classified as a non-carbohydrate food by only 45.0% of the participants, cheese by 52.5%, and cooked dried beans by 52.5%. In Domain II (carbohydrate quantification in single foods), performance was markedly lower: only 25.0% correctly estimated the carbohydrate content of one cup of pasta, 27.5% of one cup of cooked rice, and 20.0% of 100% fruit juice. Domain III (nutrition label interpretation) showed a strong performance (85.0-92.5% correct across items). In Domains IV-V (glycaemic targets and hypoglycaemia management), participants demonstrated adequate knowledge of pre-meal glucose targets (95.0% correct) and rapid treatments for hypoglycaemia (92.5%), but only 7.5% correctly identified the blood glucose increase from one carbohydrate choice. Domain VI (meal-level carbohydrate counting) was the weakest, with only 2.5% of participants correctly estimating the carbohydrate content of breakfast and lunch meals, with marginal improvement for supper (15.0%). The domain-specific performance profile is shown in **Figure 2**.

### 3.3 Diabetes distress and hypoglycaemia awareness

The mean DDS-2 score was 2.35 ± 0.90. Fifteen participants (37.5%) met the threshold for diabetes distress (DDS-2 average ≥3). The mean Gold score was 2.38 ± 1.51, with 8 participants (20.0%) classified as having impaired hypoglycaemia awareness (Gold score ≥ 4); a further 7 participants (17.5%) scored 3, the borderline value.

### 3.4 Correlations between carbohydrate counting knowledge and patient characteristics

**Figure 3:**
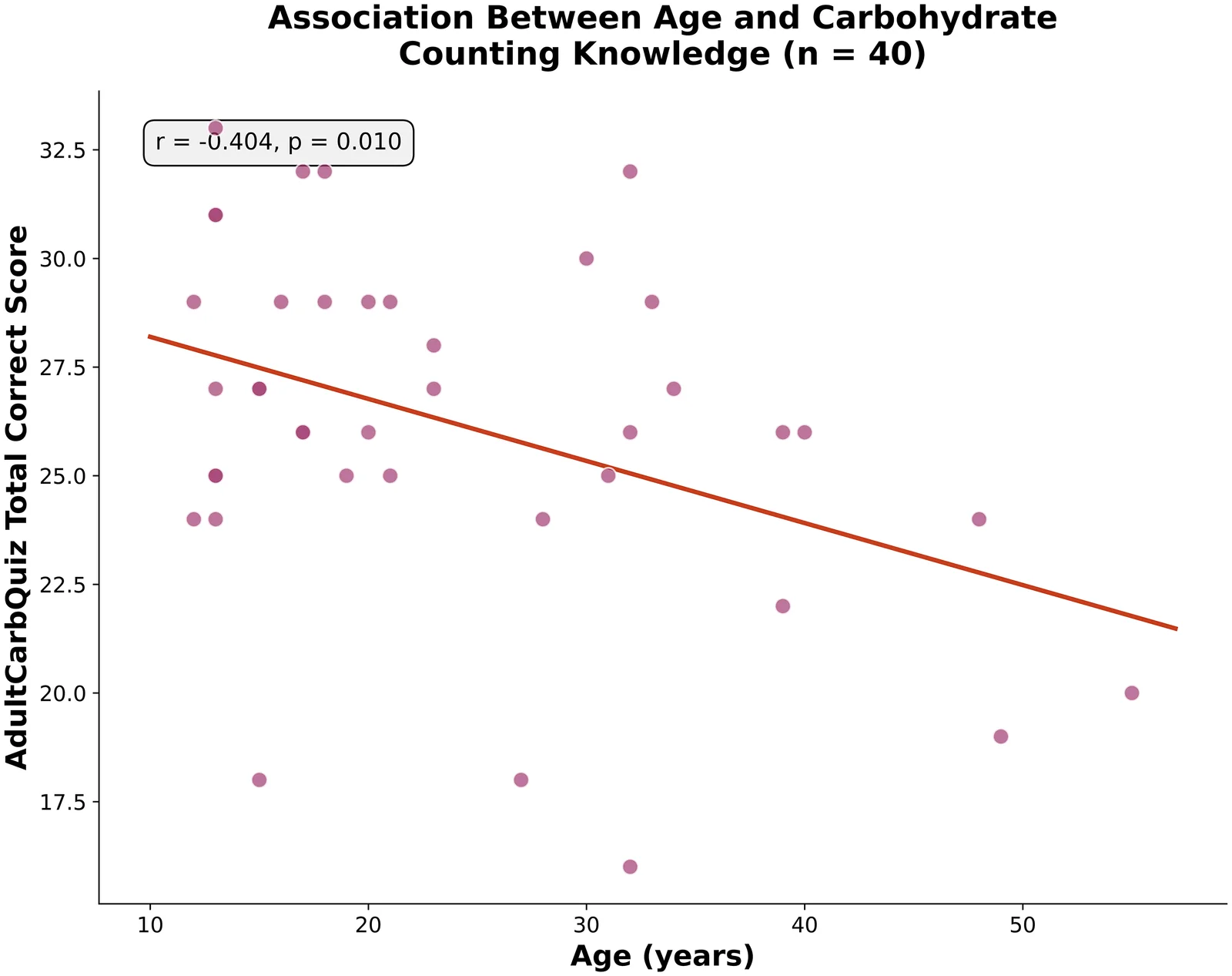
Association between age and AdultCarbQuiz total correct score (n = 40). Pearson r = −0.404, p = 0.010.

Next, we examined the association between carbohydrate counting knowledge and patient characteristics using Pearson correlations. The details are presented in **Table 2** and illustrated in **Figure 3**. Carbohydrate counting knowledge showed moderate inverse correlations with age (r = -0.404, 95% CI -0.64 to -0.11, p = 0.010), diabetes duration (r = -0.344, 95% CI -0.59 to -0.04, p = 0.030), and DDS-2 scores (average: r = -0.468, 95% CI -0.68 to -0.18, p = 0.002; item 2 [feeling of failure]: r = -0.476, 95% CI -0.69 to -0.19, p = 0.002). No associations were observed with HbA1c (r = 0.179, p = 0.295, n = 36), BMI (r = -0.187, p = 0.247), total daily insulin dose (r = 0.031, p = 0.849), or Gold score (r = -0.210, p = 0.193). In non-parametric sensitivity analyses, the associations with diabetes distress and diabetes duration were preserved (Spearman rho = -0.379, p = 0.016 and rho = -0.328, p = 0.039), whereas the association with age was attenuated and no longer significant (rho = -0.287, p = 0.072).

**Table 2:** Pearson correlations between AdultCarbQuiz total correct score and patient characteristics.

Variable	n	r	95% CI	p	Spearman ρ (p)
Age	40	−0.404	−0.64 to −0.11	**0.010**	−0.287 (0.072)
Diabetes duration	40	−0.344	−0.59 to −0.04	**0.030**	−0.328 (0.039)
Days since last CC education	40	−0.293	−0.55 to 0.02	0.067	−0.164 (0.311)
DDS-2 item 1	40	−0.342	−0.59 to −0.03	**0.031**	−0.229 (0.156)
DDS-2 item 2	40	−0.476	−0.69 to −0.19	**0.002**	−0.447 (0.004)
DDS-2 average	40	−0.468	−0.68 to −0.18	**0.002**	−0.379 (0.016)
Gold score	40	−0.210	−0.49 to 0.11	0.193	−0.194 (0.231)
Total daily insulin	40	0.031	−0.28 to 0.34	0.849	0.166 (0.305)
Background insulin	15	−0.534	−0.82 to −0.03	**0.040**	−0.466 (0.080)
Prandial insulin	17	−0.180	−0.61 to 0.33	0.489	−0.184 (0.479)
BMI	40	−0.187	−0.47 to 0.13	0.247	−0.168 (0.300)
HbA1c	36	0.179	−0.16 to 0.48	0.295	−0.033 (0.847)

Bold p-values indicate statistical significance at p < 0.05. 95% confidence intervals accompany each Pearson coefficient; Spearman coefficients are shown as a non-parametric sensitivity analysis. Background- and prandial-insulin correlations (n = 15 and n = 17) are exploratory owing to small subgroups. CC, carbohydrate counting; DDS-2, two-item Diabetes Distress Scale; Gold, Gold score; BMI, body mass index; HbA1c, glycated haemoglobin.

### 3.5 Comparisons across carbohydrate counting performance groups

**Figure 4:**
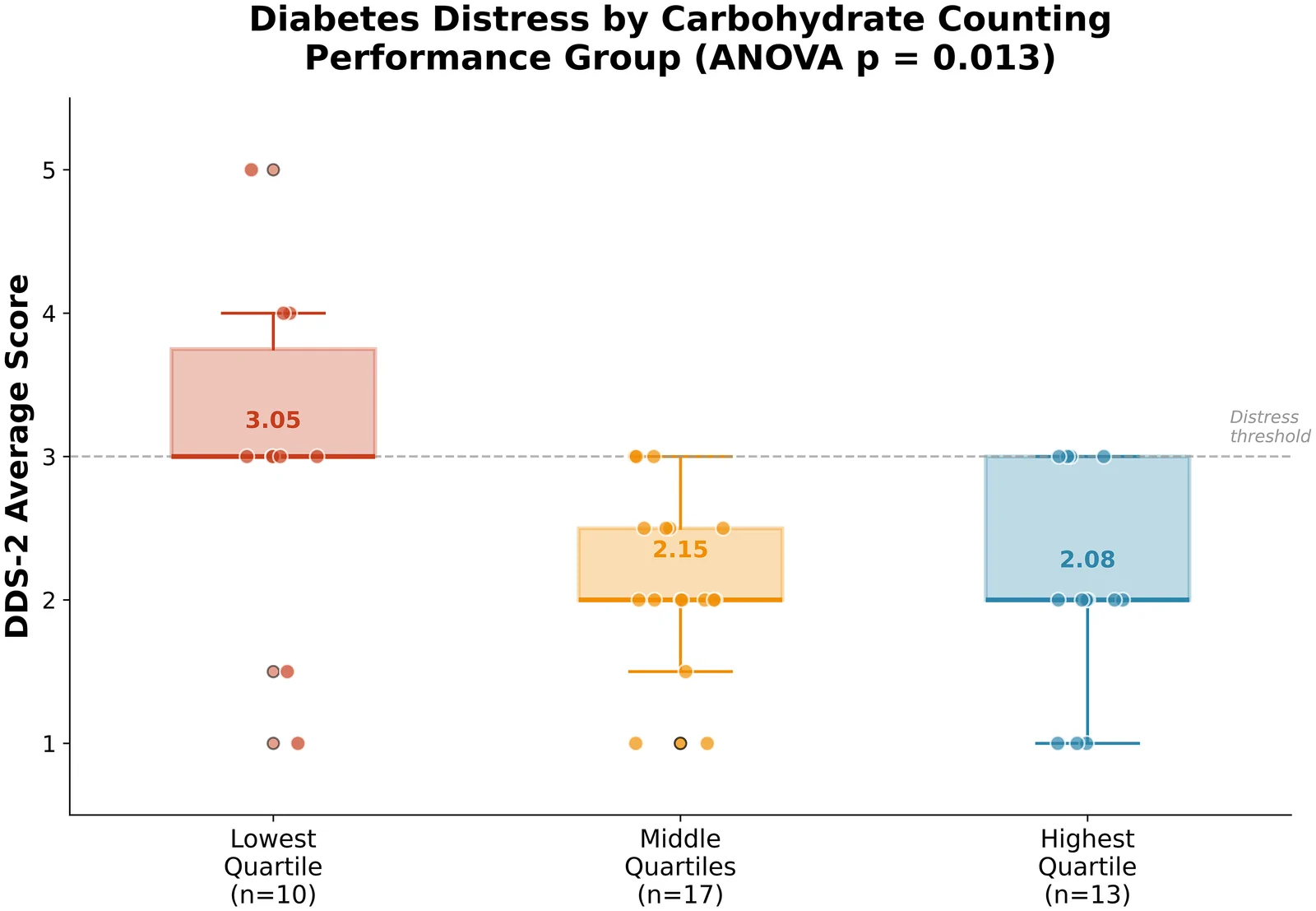
DDS-2 average scores by AdultCarbQuiz performance group: lowest quartile (n = 10), middle quartiles (n = 17), highest quartile (n = 13). One-way ANOVA p = 0.013. Dashed line indicates distress threshold.

To further examine the association between carbohydrate counting knowledge and patient characteristics, we next categorised the participants into three groups, based on total correct score quartiles (lowest quartile, middle two quartiles together and the highest quartile). These associations are shown in **Tables 3**, **4** and **Figure 4**. Participants in the lowest performance quartile tended to be older (31.8 ± 15.7 vs. 19.7 ± 7.4 years in the highest quartile), although this difference did not reach significance under Welch ANOVA (p = 0.104). They had significantly higher DDS-2 average scores (3.05 ± 1.17 vs. 2.08 ± 0.76, p = 0.013), and a higher prevalence of diabetes distress (80.0% vs. 30.8%, Fisher’s exact p = 0.004). Diabetes duration showed a consistent trend (14.1 ± 9.0 vs. 6.8 ± 7.7 years) that did not reach significance (p = 0.099). No between-group differences were observed in HbA1c, BMI, insulin doses, Gold score, nationality, or sex (all p > 0.05; **Tables 3**, **4**). Homogeneity of variance was supported for the DDS-2 comparison (Levene p = 0.43), and Tukey HSD confirmed that the lowest quartile differed from both the middle (p = 0.024) and highest (p = 0.021) quartiles. The age difference violated the equal-variance assumption and remained non-significant under both Welch ANOVA (p = 0.104) and the Kruskal-Wallis test (p = 0.202); it is therefore interpreted as exploratory.

**Table 3:** Participant characteristics by carbohydrate counting performance quartile: continuous variables.

Variable	Total (N = 40)	Lowest (n=10)	Middle (n=17)	Highest (n=13)	p (ANOVA)
Age (years)	23.98 ± 11.42	31.80 ± 15.68	22.65 ± 9.14	19.69 ± 7.40	**0.104^a^**
Diabetes duration (years)	11.05 ± 9.00	14.10 ± 9.00	12.53 ± 9.16	6.75 ± 7.72	0.099
DDS-2 item 1 (overwhelmed)	2.30 ± 0.99	3.00 ± 1.25	2.00 ± 0.79	2.15 ± 0.80	**0.029**
DDS-2 item 2 (failing)	2.40 ± 1.06	3.10 ± 1.10	2.29 ± 1.05	2.00 ± 0.82	**0.036**
DDS-2 average	2.35 ± 0.90	3.05 ± 1.17	2.15 ± 0.61	2.08 ± 0.76	**0.013**
Gold score (1-7)	2.38 ± 1.51	2.70 ± 1.57	2.47 ± 1.81	2.00 ± 1.00	0.527
Total insulin (U/day)	78.47 ± 40.51	82.50 ± 55.84	68.35 ± 26.58	88.62 ± 42.22	0.382
BMI (kg/m²)	26.00 ± 5.72	26.14 ± 4.68	27.22 ± 6.78	24.30 ± 4.84	0.391
HbA1c (%)^b^	8.06 ± 1.55	7.89 ± 1.03	7.93 ± 1.34	8.32 ± 2.04	0.769

Data are mean ± SD. Groups defined by AdultCarbQuiz quartiles: lowest (≤24, n = 10), middle (25–28, n = 17), highest (≥29, n = 13). Bold p-values indicate significance at p < 0.05. Homogeneity of variance was assessed by Levene’s test, and Tukey HSD post-hoc tests followed significant ANOVAs.

^a^Welch ANOVA. For age the equal-variance assumption was violated (Levene p = 0.026), so the Welch p-value is reported in place of the conventional one-way ANOVA value (0.030), which is not used for inference; the difference was likewise non-significant on Kruskal–Wallis testing (p = 0.202).

^b^HbA1c was available for 36 participants; group denominators differ accordingly.

**Table 4:** Participant characteristics by carbohydrate counting performance quartile: categorical variables.

Variable	Total (N = 40)	Lowest (n=10)	Middle (n=17)	Highest (n=13)	p (Fisher’s exact)
Emirati nationality (%)	87.5%	100%	82.4%	84.6%	0.491
Female gender (%)	62.5%	50.0%	82.4%	46.2%	0.074
Insulin self-administered (%)	87.5%	80.0%	94.1%	84.6%	0.581
Diabetes distress present (%)	37.5%	80.0%	17.6%	30.8%	**0.004**

Data are percentages. Groups defined by AdultCarbQuiz quartiles. P-values from Fisher’s exact test, used because expected cell counts were below five. Bold p-values indicate significance at p < 0.05. DDS-2 distress defined as average score ≥3.

### 3.6 Independent association between carbohydrate counting knowledge and diabetes distress

**Figure 5:**
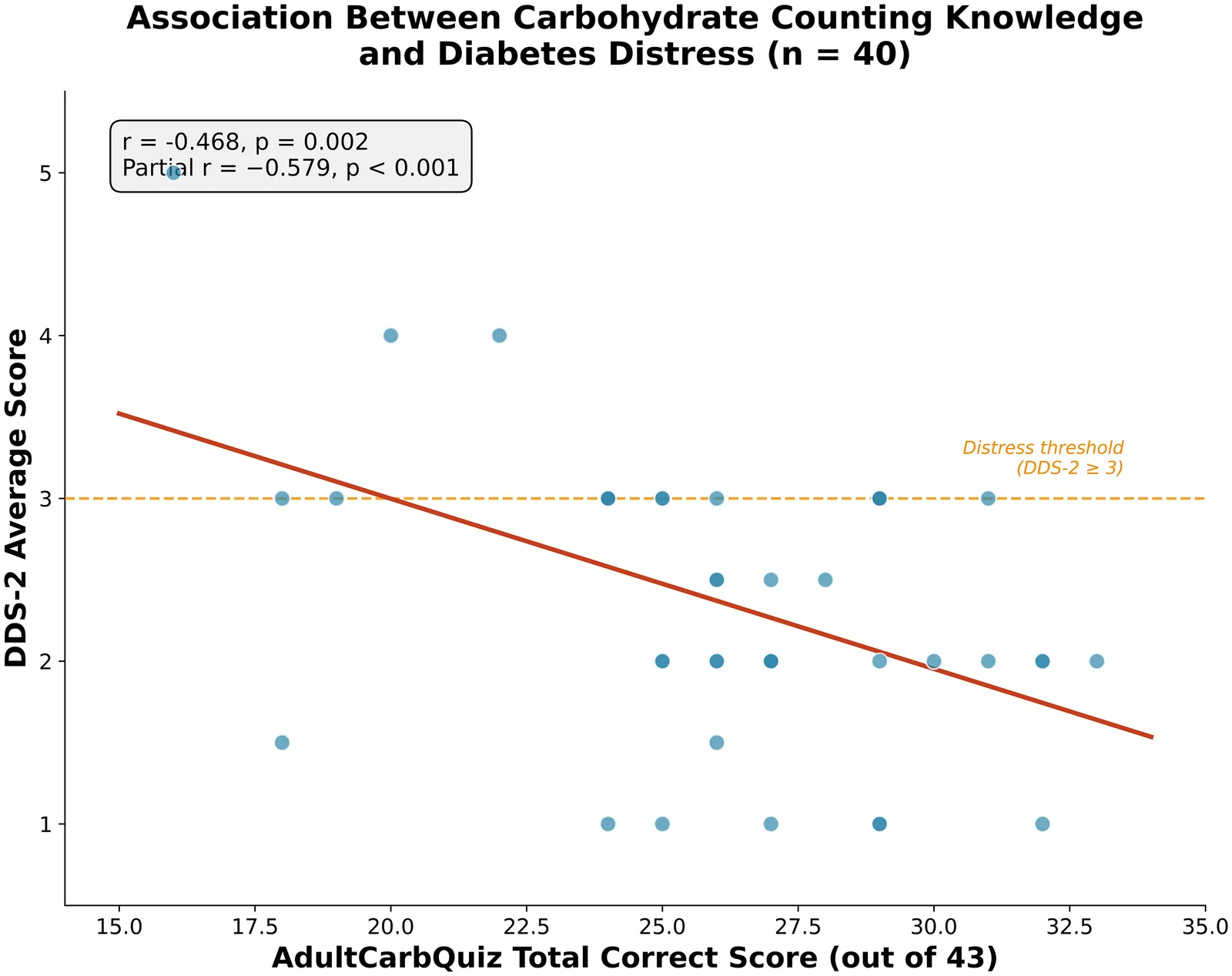
Association between AdultCarbQuiz total correct score and DDS-2 average score (n = 40). Pearson r = −0.468 (p = 0.002); partial r = −0.579 (p < 0.001) after adjustment for HbA1c and Gold score. Dashed line indicates distress threshold (DDS-2 ≥ 3).

Since there was a negative correlation between carbohydrate counting knowledge and diabetes distress, we next performed a partial correlation analysis to see if this relationship persists when adjusting for other confounders. Partial correlations adjusted for HbA1c and Gold score demonstrated that the association between carbohydrate counting knowledge and diabetes distress was strengthened (partial r = -0.579, 95% CI -0.77 to -0.30, p < 0.001), indicating that higher carbohydrate counting knowledge was independently associated with lower diabetes distress, irrespective of glycaemic control and hypoglycaemia awareness. In a sensitivity model additionally adjusting for age and diabetes duration, this association persisted (partial r = -0.425, 95% CI -0.67 to -0.09, p = 0.015). The partial correlation between carbohydrate counting knowledge and HbA1c, adjusted for the Gold score and DDS-2, remained non-significant (partial r = 0.230, p = 0.177). This association is shown in **Figure 5**. A borderline positive correlation was observed between the Gold score and DDS-2 (r = 0.306, p = 0.055), suggesting a trend towards higher distress amongst those with impaired hypoglycaemia awareness.

## 4 Discussion

To the best of authors’ knowledge, this is the first study performed to assess carbohydrate counting knowledge using a validated instrument in patients with T1DM in the UAE. The main finding of this study was that carbohydrate counting knowledge, measured by the Arabic AdultCarbQuiz, was inversely associated with diabetes distress. This association was strengthened by partial correlation analysis after adjusting for HbA1c and hypoglycaemia awareness (partial r = -0.579, p < 0.001), indicating that the knowledge-distress relationship is independent of HbA1c within this model (i.e., not confounded by glycaemic control). The study also identified a pronounced dissociation between adequate carbohydrate food recognition and poor carbohydrate quantification and meal-level counting skills, providing specific targets for educational intervention.

The mean AdultCarbQuiz score of 26.2 ± 4.0 out of 43 (60.9%) in this cohort was higher than that reported by Bawazeer et al. amongst 224 Saudi adults with T1DM (23.0 ± 7.3, 53.5%) (10) but lower than the Portuguese cohort reported by Valentim Lopes et al. (30.9 ± 3.6, 71.8%) (11). The Saudi study recruited participants from a university diabetes centre in Riyadh, where only 54% of participants used carbohydrate counting, whereas 89.7% of participants in the present study reported active carbohydrate counting use, which may partially explain the higher scores. The Portuguese cohort comprised exclusively continuous subcutaneous insulin infusion (CSII) users, a population associated with higher carbohydrate counting proficiency (11). These cross-cultural comparisons suggest that carbohydrate counting knowledge is influenced by insulin delivery modality, education frequency, and possibly dietary context.

The domain-specific analysis identified a clear hierarchy of proficiencies. Carbohydrate food recognition was strong for staple foods (bread, rice, juice: 97-100% correct) but weak for foods with ambiguous macronutrient classification, such as butter (45.0%), cheese (52.5%), and cooked dried beans (52.5%). Similar patterns of confusion between fat-rich and carbohydrate-containing foods have been reported in the original Watts validation (7) and Bawazeer et al. Saudi studies (10). The performance decline from recognition to quantification was marked: whilst most participants could identify that rice contains carbohydrate, only 27.5% could estimate the carbohydrate content of one cup of cooked rice. This gap is a key obstacle to accurate insulin bolus calculation and represents a priority target for structured educational programs that incorporate hands-on portion estimation using locally relevant foods (8, 21).

Meal-level carbohydrate counting was identified as the weakest domain, with only 2.5% of participants correctly estimating the carbohydrate content of the breakfast and lunch meals. This finding aligns with the published evidence that carbohydrate estimation errors increase with meal complexity, with the systematic underestimation of larger meals contributing to postprandial hyperglycaemia (8, 22). Practical training using real meals, visual aids, and mobile applications may address this gap more effectively than just didactic instructions (23, 24).

The inverse association between carbohydrate counting knowledge and diabetes distress was the most distinctive finding of this study. Whilst higher distress is consistently associated with poorer perceived competence and self-management in T1DM (15, 16, 25), to our knowledge few or no prior studies have directly examined the relationship between objectively measured carbohydrate counting knowledge and distress using validated instruments for both constructs. The finding that this association persists and strengthens after adjusting for HbA1c suggests that the distress-knowledge link operates through mechanisms distinct from glycaemic control. Two non-mutually exclusive explanations merit consideration. First, patients with greater knowledge may experience greater self-efficacy in dietary management, thereby reducing the emotional burden of diabetes. This interpretation is consistent with the self-determination theory framework, in which perceived competence is inversely associated with distress (15). Second, distressed patients may get disengaged from self-management education, resulting in knowledge loss. The cross-sectional design of this study cannot distinguish between these mechanisms, and longitudinal studies with repeated assessments of knowledge, distress, and glycaemic outcomes are needed to clarify the directionality.

The absence of an association between carbohydrate counting knowledge and HbA1c (r = 0.179, p = 0.295) in this cohort is consistent with several published studies (10, 11, 26). Cross-sectional correlations between knowledge and HbA1c are typically modest (r = -0.16 to -0.38) and are attenuated in populations using multiple daily injections compared with those using CSII (11). HbA1c reflects a three-month average of glycaemic control influenced by multiple behavioural, biological, and psychosocial factors beyond carbohydrate knowledge alone, including insulin adherence, physical activity, stress hormones, and intercurrent illness (27). The underpowered nature of this analysis (19% power for an effect of r = 0.18) also precludes its interpretation as a true null finding. Measures of glycaemic variability, such as time in range derived from continuous glucose monitoring, may be more sensitive to the effects of carbohydrate counting accuracy than HbA1c levels (11).

Shorter diabetes duration, and to a lesser extent younger age, were associated with higher carbohydrate counting scores, broadly consistent with the Saudi (10) and Portuguese (11) cohorts; the age association was attenuated in non-parametric analysis and should be regarded as exploratory. Bawazeer et al. reported a regression coefficient of -0.159 for age, indicating a decrease of 0.16 points per year of age (10). The mechanisms underlying this age-knowledge gradient may include more recent exposure to structured carbohydrate counting education, greater familiarity with technology-assisted dietary tools, and the transition from paediatric to adult services, where educational intensity may diminish. These findings support the need for regular knowledge reinforcement programs at periodic intervals, particularly for patients with a longer disease duration. Although our study was cross-sectional, the association we observed between carbohydrate counting knowledge and diabetes distress is consistent with interventional work linking carbohydrate counting to psychosocial as well as glycaemic benefit. In the GIOCAR randomised trial, adults with T1DM receiving continuous subcutaneous insulin infusion who were taught carbohydrate counting showed improved quality of life and reductions in BMI and waist circumference over 24 weeks, with HbA1c improving only in the per-protocol analysis (28). Commenting on the Saudi AdultCarbQuiz data, Briggs Early identified portion-size and meal-level estimation as the points at which patients most commonly falter and argued for sustained reinforcement by the wider diabetes team (29), an emphasis that our domain-level findings directly support. A programme combining group care with a carbohydrate counting curriculum has likewise reported improvements in quality of life and coping ability alongside metabolic control (30), reinforcing the case for educational models that address emotional burden as well as technical skill.

### 4.1 Strengths and limitations

This study had several strengths. To our knowledge, this is the first study to apply a validated carbohydrate counting knowledge instrument in a UAE T1DM population. The simultaneous assessment of knowledge, distress, and hypoglycaemia awareness using validated tools provides a comprehensive picture of self-management capacity. Domain-level analysis offers actionable educational targets that global composite scores cannot provide. All statistical results were independently verified using the raw dataset.

This study has several limitations. The cross-sectional design precluded causal inference regarding the distress-knowledge association. The sample size (n = 40) was adequate for the primary analysis but underpowered for detecting small effect sizes in the secondary analyses. Although the AdultCarbQuiz was validated in Arabic for a Saudi population, it includes food items based on American dietary patterns (e.g. maple syrup, hamburger patties, and dill pickles) that may not reflect the dietary context of Emirati patients consuming traditional foods such as machboos, harees, and luqaimat. The development of a culturally adapted version that incorporates local foods is warranted. The DDS-2 is a screening instrument and may not capture the full spectrum of diabetes distress assessed by the 17-item version. Our study lacked glycaemic variability data from continuous glucose monitoring, which limited the assessment of the functional impact of carbohydrate counting accuracy. The single-centre design of our study also restricts the generalisability of the results to other UAE diabetes centres. In addition, 96 of the 136 approached patients (70.6%) declined, so the enrolled cohort, of whom 89.7% already used carbohydrate counting, probably over-represents patients more engaged in their diabetes care, a potential source of selection bias. The exclusion of patients who could not read Arabic further limits generalisability to the UAE’s large non-Arabic-speaking population. No *de-novo* pilot of the instrument was conducted in the UAE. Finally, the secondary analyses were underpowered and exploratory, and the association between knowledge and age was not confirmed under non-parametric testing.

### 4.2 Clinical implications

These findings support three actionable recommendations for clinical practice: First, structured carbohydrate counting education programs should prioritise gram-based portion estimation and meal-level counting over carbohydrate food recognition, which is already adequate in this population. Second, diabetes distress screening using the DDS-2 should be integrated with carbohydrate counting knowledge assessment, as patients with elevated distress scores are likely to have lower knowledge and may benefit from combined psycho-educational interventions. Third, periodic reassessment of carbohydrate counting knowledge should be performed, particularly for patients with a longer diabetes duration, and potentially for older patients, to identify and address knowledge decay.

## 5 Conclusion

In this cross-sectional study, carbohydrate counting knowledge was independently associated with diabetes distress in patients with T1DM in the UAE, with the relationship persisting after adjusting for glycaemic control and hypoglycaemia awareness. Domain-specific analysis revealed that although carbohydrate food recognition was adequate, carbohydrate quantification and meal-level counting skills were markedly deficient in our patients. These findings support integrated clinical approaches that address both nutritional knowledge gaps and the emotional burden of diabetes self-management. Large-scale prospective studies examining whether targeted educational interventions that improve carbohydrate counting skills also reduce diabetes distress are needed.

## Data Availability

The raw data supporting the conclusions of this article will be made available by the authors, without undue reservation, subject to approval by the Tawam Hospital Research and Ethics Committee and in accordance with applicable patient confidentiality requirements.
